# PROMs and PREMs in routine perinatal care: mixed methods evaluation of their implementation into integrated obstetric care networks

**DOI:** 10.1186/s41687-023-00568-w

**Published:** 2023-03-09

**Authors:** Anne L. Depla, Bettine Pluut, Marije Lamain-de Ruiter, Anna W. Kersten, Inge M. Evers, Arie Franx, Mireille N. Bekker

**Affiliations:** 1 Department of Obstetrics and Gynecology, Wilhelmina Children’s Hospital, University Medical Centre Utrecht, KE.04.123.1, Lundlaan 6, 3584 EA Utrecht, The Netherlands; 2 Erasmus School of Health Policy & Management, Erasmus University Rotterdam, Rotterdam, The Netherlands; 3 Department of Obstetrics and Gynecology, Erasmus MC Sophia, Rotterdam, The Netherlands; 4 Department of Public Health, Julius Centre, University Medical Centre Utrecht, Utrecht, The Netherlands; 5 Department of Obstetrics and Gynecology, Meander Medical Centre, Amersfoort, The Netherlands

**Keywords:** Perinatal care, Patient-reported outcome measures, Patient-reported experience measures, Implementation science, Action research, Value-based healthcare

## Abstract

**Background:**

In the transition towards value-based healthcare, patient-reported outcome and experience measures (PROM and PREM) are recommended by international collaborations and government programs to guide clinical practice and quality improvement. For many conditions, using PROM/PREM over the complete continuum of care requires implementation across care organizations and disciplines. Along PROM/PREM implementation in obstetric care networks (OCN), we aimed to evaluate implementation outcomes and the processes influencing these outcomes in the complex context of care networks across the continuum of perinatal care.

**Methods:**

Three OCN in the Netherlands implemented PROM/PREM in routine practice, using an internationally developed outcomes set with care professionals and patient advocates. Their aim was to use PROM/PREM results individually to guide patient-specific care decisions and at group-level to improve quality of care. The implementation process was designed following the principles of action research: iteratively planning implementation, action, data generation and reflection to refine subsequent actions, involving both researchers and care professionals. During the one-year implementation period in each OCN, implementation outcomes and processes were evaluated in this mixed-methods study. Data generation (including observation, surveys and focus groups) and analysis were guided by two theoretical implementation frameworks: the Normalization Process Theory and Proctor’s taxonomy for implementation outcomes. Qualitative findings were supplemented with survey data to solidify findings in a broader group of care professionals.

**Results:**

Care professionals in OCN found the use of PROM/PREM acceptable and appropriate, recognized their benefits and felt facilitated in their patient-centered goals and vision. However, feasibility for daily practice was low, mainly due to IT issues and time constraints. Hence PROM/PREM implementation did not sustain, but strategies for future PROM/PREM implementation were formulated in all OCN. Processes contributing positively to implementation outcomes were internalization (understand the value) and initiation (driven by key-participants), whereas challenges in relational integration (maintain confidence) and reconfiguration (refine activities) affected implementation negatively.

**Conclusion:**

Although implementation did not sustain, network-broad PROM/PREM use in clinic and quality improvement matched professionals’ motivation. This study provides recommendations to implement PROM/PREM meaningfully in practice in ways that support professionals in their drive towards patient-centered care. In order for PROM/PREM to fulfill their potential for value-based healthcare, our work highlights the need for sustainable IT infrastructures, as well as an iterative approach to refine their complex implementation into local contexts.

**Supplementary Information:**

The online version contains supplementary material available at 10.1186/s41687-023-00568-w.

## Background

In the past decade, the discourse of value-based healthcare (VBHC) has had an immense uptake in healthcare [1]. At system level, healthcare systems strive to use patients’ well-being to evaluate care performance for full treatment cycles for a condition [2]. At patient level, professionals aim to organize integrated care around a health condition and make personal values prescriptive to guide treatment decisions [3]. In the development towards VBHC, patient-reported outcomes and experiences measures (PROM and PREM) have been embraced to generate data about what matters to patients and drive patient-centered quality improvement (QI) [4]. Therefore, the capture and use of PROM/PREM has been encouraged in many healthcare settings by international collaborations and government programs [5, 6]. Nevertheless, PROM/PREM implementation remains challenging, especially in network settings like perinatal care [7, 8].

PROM/PREM implementation has been considerably studied with an implementation science approach, identifying common influencing factors such as technology and clinical leadership [9, 10]. Different challenges have been described dependent on the purpose of PROM/PREM implementation. For example, a challenge for individual-level use includes fitting PROM collection to appointment schedules, while at group-level motivating care professionals for (external) QI appears more challenging [8, 9]. Yet most implementation factors have been explored in single organization settings or primary care predominantly [11, 12], whereas the majority of health conditions require interdisciplinary and interorganizational collaboration across healthcare tiers to provide the full continuum of care [13, 14]. As for pregnancy and childbirth, where care professionals collaborate both interdisciplinary (e.g., obstetrics, neonatology) and interorganizational (e.g., hospitals, midwife practices, youth care) to provide acute and long-term care with in-hospital, outpatient and community-based care and support. Thus, to contribute to patient- and family-centered care, PROM/PREM in perinatal care would ideally be implemented across care networks, to cover patients’ whole care trajectory in individual-level use and involve all stakeholders in group-level use for QI. Yet, implementation in network context prompts other challenges, like engaging diverse stakeholders, aligning incentives and resources, and building common infrastructures [4, 15]. Evaluations of individual-level PROM/PREM implementation in network context are scarce, but needed to advance our understanding of practice challenges, contextual factors, and mechanisms through which implementation strategies work across organizations [10, 16].

For perinatal care, until recently, no consensus on PROM/PREM had been formed to evaluate its patient outcomes [17]. Yet, in 2017, a set of standardized patient-centered outcomes measures for pregnancy and childbirth (PCB set) was developed internationally with perinatal care professionals and patient advocates [17, 18]. This set includes PROM/PREM from beginning of pregnancy until six months postpartum. Over the last years, the PCB set has been adopted internationally and implementation efforts have been started worldwide, of which most are in research context [19–21]. Potential factors influencing PCB set adoption in practice have been explored in pre-implementation analyses, indicating all stakeholders recognized the relevance and potential benefits of PROM/PREM [8, 22]. At the same time, stakeholders acknowledged important efforts yet to be made, e.g., embedding PROM/PREM into service processes or informing care professionals and patients about their purpose.

Recently, the patient-reported measures of the PCB set were implemented in three obstetric care networks (OCN) in the Netherlands, that aimed to use these PROM/PREM for two levels of VHBC: individual scores to guide patient-specific care decisions and group-level results in to improve quality of care. This implementation process was designed following the principles of action research to enhance practice change and, concurrently, gain knowledge about PROM/PREM implementation in the context of care networks. Guided by theoretical frameworks for implementation, this study aimed to evaluate (1) the outcomes of PROM/PREM implementation in obstetric care networks and (2) the implementation processes that influence these outcomes to increase our understanding of this complex implementation, its practice challenges, and underlying change mechanisms.

## Methods

### Design

This mixed-methods study was conducted between December 2019 and June 2022 as part of an action research project aimed at PROM/PREM implementation in clinical practice and QI processes of OCN. Action research aims to both change practice and develop knowledge about that change via a cyclic design of action, data generation and reflection, while involving all stakeholders in research and practice change [23]. Action research is particularly useful to implement a complex intervention that needs adjustment to the local context, as detailed data are generated on both the implementation activities (what it involved) and change mechanisms (how it worked). This way, the outcomes achieved can be explained for, increasing the transferability of findings [24]. To understand the change mechanisms underlying the complex implementation of PROM/PREM, the use of multilevel implementation frameworks and theories has been recommended by scoping literature [10, 25]. To evaluate PROM/PREM implementation in the context of care networks, this study combinedly used Proctor’s taxonomy for implementation outcomes [26] and the Normalization Process Theory (NPT) [27]. Proctor’s taxonomy describes the outcomes of different stages in implementation, whereas the NPT describes implementation processes in terms of what care professionals (don’t) do to embed a new way of working in routine practice and is distinct in proposing mechanisms for sustained uptake. Proctor and NPT guided the collection and analysis of both qualitative and quantitative data within the mixed-methods design, increasing both the depth and transferability of our findings.

### Setting and participants

Dutch perinatal care is provided interdisciplinary from two healthcare tiers: primary care by community midwives and maternity care organizations; and secondary/tertiary care by hospital employed care professionals. Hospitals, regional community midwife practices and maternity care organizations increasingly cooperate in OCN to provide continuity of care across pregnancy, childbirth and puerperium. In 2019, PROM/PREM implementation was initiated from a regional collaborative between ten OCN in the middle of the Netherlands, of which three OCN participated. In each OCN, the hospital and 2–4 midwifery practices implemented individual-level PROM/PREM in clinic. All other professionals working in the OCN (e.g., from other midwifery practices, maternity care organizations, youth care) could join network-broad QI with group-level outcomes. Each OCN had an interdisciplinary team in charge of implementation (including, at least one obstetrician, clinical midwife, and community midwife from each participating midwifery practice), of which one was appointed project leader. In this study, participants were defined as (1) professionals *directly* involved in implementation: project team members (key participants) or obstetricians/midwives using individual-level PROM/PREM, and (2) *indirectly* involved professionals: from other OCN-organizations or discipline, such as nurses. Patients were involved in implementation as they completed PROM/PREM for routine care but did not actively participate in this evaluation study. As patients had participated in our pre-implementation analysis and feasibility pilot [8, 28], their needs were incorporated in the initial implementation strategy.

### Action research project

**Fig. 1:**
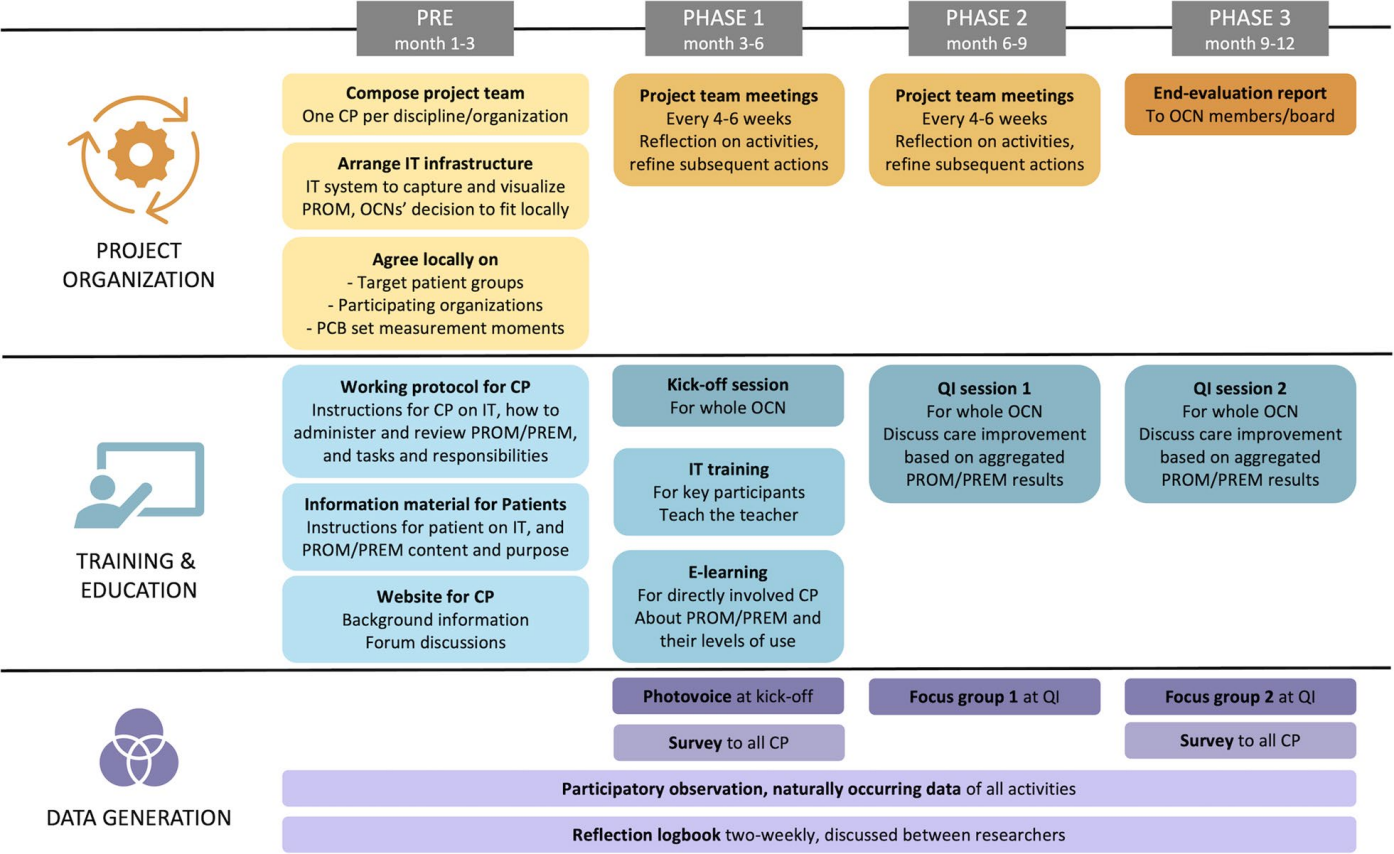
Timeline of implementation and data generation activities. *PROM*, patient-reported outcome measure. *PREM*, patient-reported experience measure. *QI*, quality improvement. *OCN*, obstetric care network. *CP*, care professional. *VHBC,* value-based healthcare

The PROM/PREM implemented in this project were those proposed in the PCB set: questionnaires at two moments during pregnancy (T1: first trimester, T2: early third trimester) and three postpartum (T3: maternity week, T4: 6 weeks postpartum, T5: 6 months postpartum). The PCB set was developed internationally and subsequently translated to the Dutch setting, both phases involving all stakeholders, including care professionals and patients [18, 29]. An overview of the PCB set’s patient-reported domains and timeline for completion is provided in Additional file 1: Fig. S1. The set’s PROM/PREM were implemented for two purposes. First, individual-level PROM/PREM were implemented in clinic: reviewing N = 1 scores with patients during a regular care contact after completing a questionnaire. The timeline of collection, workflow, and follow-up services (including scoring and alert values) were organized as described in the national pilot project [30]. Second, the same PROM/PREM outcomes would be used at group-level in network-broad QI sessions. Despite the complexity of combining these purposes, findings in our pre-implementation research amongst care professionals, patients and other stakeholders in perinatal care suggested both goals could also reinforce each other [8]. Direct usability in clinical practice could, for instance, motivate care professionals and patients to comply, thereby generating data for group-level use (and vice-versa). Likewise, other previous findings from our pre-implementation analysis and feasibility pilot [8, 28], were used to design the initial implementation strategy. Important elements for individual-level use included visual alerts to support care professionals in interpreting the answers and offering patients a choice whether their care professional had insight in their individual PREM answers. During the action research project, this initial implementation strategy (Fig. 1) was continuously refined guided by action research principles in iterative cycles of planning and executing implementation activities, data generation, and reflection on these data to refine subsequent activities. These cycles were conducted jointly by researchers and care professionals. The researchers developed the baseline strategy for project organization and education (e.g. identified possible IT-systems, developed an e-learning and kick-off meeting), provided materials and support for its execution (e.g. patient information folder, for working protocol for care professionals), and facilitated data generation for its refinement (e.g. organized focus groups, sent out the survey). The project teams designed and coordinated local implementation (e.g. adapt instruction material to local workflow, chose the IT system that best fitted local needs and resources) and participated in data generation and reflections (e.g. survey results were discussed in project team meetings, participation in focus groups). Three OCN started implementation sequentially to be able to learn from previous experiences, exchanged via the researchers and directly between care professionals from different OCN. After the one-year implementation period, project teams reported their experiences to their OCN and advised future steps in an end-evaluation.

### Outcome measures

First, implementation outcomes were assessed using Proctor’s taxonomy of implementation outcomes. Inspired by the translation to PROM/PREM specific implementation outcomes by Stover et al. [10], implementation outcomes and the indicators to assess them were defined for this study’s context (Table 1). These indicators were evaluated with survey items of the Measurement Instrument for Determinants of Innovations (MIDI), via administrative data and embedded in qualitative methods such as observation checklists. The MIDI was developed to identify factors influencing the use of an implemented intervention by measuring determinants in innovation, user, organization and socio-political context [31]. As recommended by its developers, a selection of items was made based on relevance for our context. Second, implementation processes were evaluated along the NPT, which describes four core mechanisms towards normalization. These mechanisms and their subconstructs were measured trough the validated Normalization Measurement Development (NoMAD) instrument [32, 33], and were included in the survey and qualitative methods (Additional file 1: Table S1). The complete survey administered to care professionals consisted of validated NoMAD and MIDI items, completed with three extra questions (about education used, knowledge level, needs in implementation) based on our feasibility pilot and PROM/PREM specific implementation literature [28, 34]. All survey questions and details about scoring are provided in Additional file 1: Table S2.

**Table 1:** Implementation outcomes and their assessment

Implementation outcome	Definition	Indicators	Assessment methods
Acceptability	Perception among CP that the PROM/PREM are agreeable, palatable, or satisfactory	Expected relative advantage Expected reporting ease/comprehensible PROM/PREM and IT system	Qualitative^a^ Observations^b^ Survey (MIDI 8 and 15; Extra 3)
Adoption	Initial decision to implement the PROM/PREM	Participating hospitals and midwifery practices Representativeness of those clinics; reason to participate	Administrative data Observation
Appropriateness	Perceived fit, relevance, or compatibility of the PROM/PREM for a) midwifery practices, hospitals, CP, pregnant women, and b) their goal to guide personal care and quality improvement	PROM/PREM fit patient (level, language, condition, font size) PROM/PREM fit professional (visualized, easy access, decision support) PROM/PREM fit culture and values (leadership support) PROM/PREM fit goals: helpful to discuss symptoms/improve care	Qualitative Observations Survey (MIDI 9, 12, 26)
Feasibility	Extent to which PROM/PREM can be successfully used or carried out within the OCN, midwifery practice, hospital	IT: technical issues, adaptability to visualize PROM/PREM meaningful Usability for patients (access, timing) Usability for professional (time efficiency; capable; support)	Survey (MIDI 13 and 16; Extra 1) Qualitative Observations
Fidelity	Degree to which PROM/PREM were implemented as described originally	Consistency of administering PROM/PREM Professionals reviewing PROM/PREM results with patients How and why local adaptations (time points, patient groups)	Observations Administrative data
Implementation cost	Cost impact of the effort to implement PROM/PREM	Technology costs Personnel and time	Administrative data Observations; Qualitative
Penetration	Integration of PROM/PREM in OCN, midwifery practices and hospitals	Targeted patient groups Professionals: involved (or knowledge), training attendance	Survey (MIDI 18 and 28; Extra 2) Observations; Qualitative
Sustainability	Extent to which the PROM/PREM are maintained within an OCN, midwifery practice or hospital	Normalization/routinized (carry on; with what?) Stakeholder perceptions	Administrative data Observations; Qualitative

*OCN* obstetric care network, *CP* care professional, *PROM* patient-reported outcome measures, *PREM* patient-reported experience measures, *IT* information technology, *MIDI* Measurement Instrument for Determinants of Innovations

^a^Qualitative methods: indicators were embedded in coding schemes of all qualitative data (i.e., open-ended survey answers, transcriptions, observation reports, reflection logbook, naturally occurring documents)

^b^Observations: performed along a checklist with these indicators while participating in implementation activities (i.e., project team meetings, kick-off sessions, QI sessions and two-weekly reflection logbook)

### Data generation

A timeline of data generation is provided in Fig. 1. For quantitative data, the survey was sent to all OCN care professionals at the start and end of implementation by e-mail. Care professionals indirectly involved in implementation were led to a short version. Demographics were collected on profession and working experience. This way, the survey explored implementation processes in a broad group of care professionals, which was used to solidify qualitative findings and to guide reflection on the implementation process and needs with participants during qualitative methods. Qualitative data were generated through focus group discussions, observations, reflections and naturally occurring data. At each kick-off session, group discussion was organized using photovoice (i.e. a method to empower all participants to share their perspectives [35]), of which notes were taken for the observation report. Along the QI sessions, traditional focus group discussions were led by two researchers (AD, AK) along statements about implementation based on outcome indicators and NPT subconstructs (Additional file 1: Table S3). For each focus group, a selection of these statements was made to address specific gaps in data generation emerging from collective iterative reflections and quantitative results from the survey. After informed consent, focus groups were recorded and transcribed ad verbatim. During the whole implementation, two researchers (AD, ML) conducted participative observations in all meetings and kept a reflection logbook, both structured along the theoretical frameworks. Considered as naturally occurring data [36], all documents emerging during the implementation process were gathered (e.g., meeting reports), containing administrative data too (e.g., IT system data on costs, professionals with account).

### Data analysis

Quantitative survey data were analyzed in R version 4.0.2 [37]. Mean scores were calculated for items consisting of multiple statements and multiple items measuring a subconstruct. Frequencies of responses to items were visualized in stacked-bar diagrams to gain insights in the diversity of opinions. All qualitative data (i.e., open-ended survey answers, transcriptions, observation reports, reflection logbook, documents) were thematically analyzed in Microsoft Excel version 16.61 conform QUAGOL guidelines, combining a deductive and inductive approach [38]. The researchers assigned codes from the conceptual frameworks (Proctor and NPT) as well as open codes describing themes within their concepts. At start, three researchers (AD, BP, ML) coded three documents independently, and discussed the resulting codes to develop a mature coding scheme. Data were then analyzed by AD until saturation was reached, after which four researchers (AD, ML, BP, MB) reviewed and discussed the codes to establish final interpretations. Quantitative and qualitative data were then triangulated by exploring (dis)agreements and silences between both datasets. This was conducted by a single researcher (AD) identifying items and subconstructs in the quantitative dataset demonstrating particularly high or low survey scores, to compare these against qualitative themes and discuss that among the research team. In this process, quantitative data were used to solidify quantitative findings in a broader group of professionals and over time.

## Results

Overall, 159 surveys were returned, of which 63 (39%) in phase 1 and 97 (61%) in phase 3. Five focus groups were held with, in total, 78 care professionals attending QI sessions. Other data (from observations, reflections, documents) were generated along 39 project team meetings, 3 kick-off sessions, 5 QI sessions, and the logbook. Participants’ characteristics for the survey and focus groups are presented in Table 2. Of survey respondents, 62% (99/159) was directly involved in implementation (i.e., project team member or using individual-level PROM/PREM). Mean survey scores were largely in agreement with qualitative themes, thus strengthening each other, and are together presented per theoretical framework below. Full response frequencies per survey item are provided in Additional file 1: Fig. S2.

**Table 2:** Baseline characteristics survey and focus group participants

Characteristic	Survey, N = 159	Focus groups, N = 79
*Profession*		
Community midwife	64 (40%)	39 (49%)
Hospital midwife	27 (17%)	14 (18%)
Obstetrician/gynecologist	17 (11%)	10 (13%)
Obstetric resident	11 (7%)	9 (11%)
Obstetric nurse	21 (13%)	4 (5%)
Maternity care	13 (8%)	2 (3%)
Neonatologist/pediatrician	2 (1.3%)	0
Youth care professional	1 (0.6%)	1 (1%)
Other^a^	3 (2%)	–
*OCN region*		
OCN 1	55 (35%)	11 (14%)^b^
OCN 2	46 (29%)	34 (43%)
OCN 3	58 (36%)	34 (43%)

*OCN* obstetric care network

^a^Managers, n = 2. Missing, n = 1

^b^In OCN 1, just one focus group was held so a community midwife was interviewed here in phase 3 (month 9–12 of implementation)

### Implementation outcomes

Below, Proctor’s outcomes as defined in Table 1 are provided along our most important findings.

#### Motivations and objectives

At the start, potential benefits of PROM/PREM were recognized by most care professionals, contributing to *acceptability* and *adoption.* Care professionals expected that individual-level PROM/PREM would assist them in recognizing symptoms and identifying topics important to their patient and empower patients to prepare visits and raise issues. Moreover, care professionals expressed enthusiasm for using group-level PROM/PREM for patient-centered quality improvement. Patients’ opinions were care professionals’ main motivation to comply and 54% (46/85) of survey respondents expected their cooperation, whereas 11% (9/85) did not.

#### Experienced benefits

According to care professionals, system-wide PROM/PREM capture and use facilitated their patient-centered goals and vision, expressing good *appropriateness*. In consultations, several care professionals felt supported by PROM/PREM results to identify and discuss patients’ issues, sometimes leading to richer conversations and/or appropriate referrals. From the group-level PROM/PREM data in QI sessions, care professionals gained valuable insights and directions for improvement in their patients’ wellbeing and experiences, which contributed to their work pleasure. In practice, the PROM/PREM content was considered *appropriate* for most of their patients, except for non-Dutch speaking women and those with low health literacy, who care professionals hesitated to invite for that reason. Also, some adaptations to PROM/PREM content were suggested, such as open answer options to enable personalized care even more.

#### Experienced barriers

Whilst most care professionals strongly favored integration in their electronic health record (EHR), the locally explored IT options either could not function across different EHRs, or their costs to realize that were too high. Hence, in each OCN, an affordable start-up IT system without EHR integration was chosen, that promised automated data capture, visualization for care professionals, network communication and privacy. This IT system enabled PROM/PREM *adoption* in all participating practices per OCN but became the main barrier for *acceptability, feasibility,* and further implementation. Care professionals did not consider it to be user-friendly (complicated access, frequent issues and bugs, poor overview, not visible whether responses had been discussed and unable to connect PROM/PREM measurements to visits) and felt increasingly frustrated by the IT supplier’s slow pace, and sometimes inability, to solve issues. Although their patients often appeared willing to complete PROM/PREM, IT was considered a major barrier for patients too, due to poor accessibility and bugs, leading to privacy concerns too. Other patient barriers mentioned were a lack of motivation or time (especially postpartum) and misunderstanding of the purpose.

Additionally, the high time investment for care professionals negatively influenced *acceptability* (44% of care professionals (60/135) expected it would take too much time), *appropriateness* (for their high current workload) and *feasibility* (of workflow integration). Factors contributing to a high time investment in practice included the administrative burden of the non-integrated IT system, instructing patients, reviewing PROM/PREM results, and learning a new skill.

#### Costs

The IT systems’ costs and care professionals’ time investment (i.e., project team efforts and using PROM/PREM in practice) were the main drivers for *implementation costs*. In two of three OCN, these costs demanded external funding (used for the IT system and project leader allocation); the third OCN could finance them from a joint reimbursement structure.

#### Fidelity and penetration

The process of creating an account for the external IT system, inviting patients, and discussing individual PROM/PREM responses required continuous support from project teams and action researchers to reach *fidelity* and *penetration* in participating practices. At start, project teams decided to begin with a selection of patient groups, measurement moments (all selected T1–T4) and care professionals. Eventually, most targeted care professionals created an IT system account, but only few actively invited patients: others often missed eligible patients due to time constraints and low exposure resulting from the patient group selection. Half of the invited patients created an account and completed PROM/PREM; postpartum response rates were lower. Based on experiences shared in project team meetings, almost all completed PROM/PREM were discussed in the next visit, except in case of IT bugs or care transitions in the maternity week (T3). Regarding group-level PROM/PREM use, five QI sessions were carried out during the pilot periods. Reflecting good *fidelity,* local care professionals actively participated in preparation, presentation, and elaboration of these sessions, which were attended by an average of 17 (range 11–25) care professionals representing all participating disciplines. Unlike in-clinic PROM/PREM use, QI sessions extended *penetration* to care professionals without direct involvement in implementation.

#### Sustainability

Except for one community midwifery practice that *sustainably* integrated PROM/PROM, routine PROM/PREM administration was stopped in all OCN after the one-year implementation period. However, all OCN intended to continue the QI sessions with data available in the OCN and, after EHR integration, reinitiate PROM/PREM capture and use. After the decision to stop, the second QI session in one OCN was not conducted, because the project team expected it would be of more benefit to a future restart.

### Implementation process

The complete NPT framework analysis is listed in Table 3 with supportive qualitative and quantitative data (mean survey scores on a 5-point Likert scale) per subconstruct. Per core mechanism, subconstructs contributing most to (un)successful implementation outcomes are elaborated on below. Overall, main processes contributing to implementation positively were *internalization* (understand value) and *initiation* (drive by key-participants), whereas *relational integration* (maintain confidence) and *reconfiguration* (attempts to redefine) affected implementation negatively.

**Table 3:** Framework analysis for the NPT subconstructs with supportive data

NPT mechanisms and subconstructs	Framework analysis	Illustrative quotes and observations
*Coherence* *“Sense-making work as individuals and collective”*		
Differentiation	Most CP able to differentiate PROM^a^ from normal work Terminology (ICHOM, VBHC, PROM) made it abstract Helped by previous experiences, kick-off session, e-learning	*PTM1, OCN 3, HM/PL(OCN2)*: [preparing kick-off session, PL of OCN2 attends PTM to help] “I would present the practical aspect of discussing PROM answers, along an example case. Otherwise, it remains quite abstract with the ICHOM circle and value-based healthcare”
Communal specification	Shared vision amongst kick-off and QI session participants: goal is patient centered care and collaboration for better outcomes Varied per OCN if staff had aligned project goals and whether their goal was clear to CP in clinic Easier in OCN with a more mature organization structure	*PTM18, OCN1, report:* [after doubts in previous meeting] “Looking back, participation of X (the hospital) and X (a midwifery practice) in this project has surely been discussed and decided upon in the OCN. They would start with the PROM and evaluate whether it’s feasible to move up with the whole OCN and would report that to the OCN”
Individual specification	Most CP comprehended individual tasks, supported by the protocol, IT training and key participants PROM often interpreted as research: both CP and patients	*PTM18, OCN1, observation:* “She (CM), and the other project team members, have always interpreted the PROM as research and informed patients that way too. […] Now, she informs her patients clearer that PROM completion is for their own care and, thereby, in their own good. She states that she gets more responses and has to ‘go after it’ less. That is also really motivating for themselves (CM + her colleague)”
Internalization	Most CP constructed potential value of PROM at start Helped further by e-learning, kick-off session and previous experiences Not all CP aware of ownership to use group-level PROM for QI	*QI1, OCN3, mentimeter*: [after introduction video: what attracts you in the video?] (Attending CP) “Honest answers, ability to raise issues, more fulfilment of work, elaboration of a person, customized care, patient at the center, being seen, prepared for situation, personal attention”
*Cognitive participation* *“Relational work to build and sustain a community of practice around the intervention”*		
Initiation *Mean survey score*^b^*: 3.82*	Project leader and team members initiated with training and support. These key-participants felt facilitated by one-year implementation period (felt as ‘try out’) and by the action research project providing materials, support and earlier regions experiences Still, for some CP, it felt the PROM appeared without explanation Towards end: key-participants stopped motivating their peers, feeling they asked too much effort whilst IT issues were unresolved	*Kick-off, OCN2, observation:* PL takes charge in the presentation, including general information and vision why the project is carried out. […] The project team members are clearly the early adopters/key-participants, also clear for the other CP attending this kick-off session. The project team members have completed the e-learning and the IT training already {before this kick-off session)”
Enrolment *Mean survey score: 3.84*	Most CP open to working with PROM, some wanted to await results Sceptic/hesitant about technology, time investment, patient burden Helped by education/training, but continuous attention and support in practice more important regarding low training attendance Impaired by little real-life contact (COVID-19 pandemic) Harder in larger organizations with distant leadership	Kick-off, OCN2, observation: [question attitude towards PROM]: Most CP answer positive, few neutral, no negative. CPs answering positive share they have a better understanding of what is expected from them. They praise the project team for their good preparations and hard work. CP answered neutral because of worries about IT, workload, and uncertainty about the exact time investment”
Legitimation *Mean survey score: 3.48*	Most midwives and obstetricians considered PROM a legitimate part of their role (except T5). Others felt not in need of PROM to discuss the topics these PROM address: felt as check/registration burden CP without active involvement invited to QI sessions (i.e., obstetric nurses, preventive youth care, non-participating CM) had to attend before understanding their valid contribution	*Interview, OCN1, CM*: “It somewhat has been brought, in my experience, like “well this is really the tool to provide personal care”. […] In my opinion, I already provide very personal care and all freedom for women to feel the opportunity to raise their personal items. […] And yes, then we [CP] get the next check off list on our plates”
Activation *Mean survey score: 3.69*	Over time, IT issues for both CP and their patients created resistance.—Most CP stopped working with PROM because too much effort and time (mainly IT, see feasibility) for little gain (low PROM exposure) Key-participants and OCN boards continued to support the potential of PROM for VBHC and looked for alternatives to embed them	*Focus group, OCN2, multiple CP*: "(CM3) yes but the question… the content of the project: that was something we fully supported. Well, I’ll speak for myself, I fully supported that. Only how the IT system… that was where it got stuck on for me. (CM2) well, for everybody here (OB) also for our patients”
*Collective action* *“Operational work to enact a set of practices”*		
Interactional workability *Mean survey score: 2.91*	Protocol and experiences form earlier regions helped. Still, hard to integrate PROM in clinical routine (IT issues, time) Different experiences of CP how long the PROM took to discuss, but existing workload was already high, with little time to learn new skills CP needed more exposure to build routine. Yet local project teams hesitant with expansion because of time investment and IT struggles	*Evaluation report, OCN2, CM*: “However, the usability [of the IT system] causes irritation, both in midwives as well as patients. Our patients complain about leaving personal data, the barrier to log-in and recurrent reminders even if they already completed the PROM. For midwives, integrating PROM in their consultations remained difficult, having to log-in to an external system is a barrier”
Relational integration *Mean survey score: 3.71*	ROM were mostly an individual task in clinic, highest workload CM Group-level PROM results led to conversations about improvement opportunities in the OCN, motivating CPs’ implementation efforts and contributing positively to working culture and pleasure Trust in the innovation was negatively affected by bad functioning IT system, privacy questions and PROM content or timing	*PTM18, OCN1, report*: “She (HM) also states that she feels all negative emotions about the use of the IT system also affect CP’s receptivity for the idea of value-base care” *Evaluation report, OCN2, HM/PL:* "A hindering factor for CP was the uncertainty whether questionnaires were sent out. Sometimes they would not be sent out at all, and it wasn’t clear to the CP whether this was due to the IT system or a problem in patient’s registration”
Skill set workability *Mean survey score: 3.41*	Most CP felt skilled to use PROM in clinic and for QI. CP negative on self-efficacy, CP needed more time, administrative staff, open answer options to the PROM, and better IT and data-analysis Allocating administrative tasks was difficult because of the external IT system (e.g., manual tasks: enter delivery date, invite patients)	*PTM8, OCN1, PL*: “After birth, date of delivery has to be entered in the IT system directly to send out the postpartum questionnaires on time. The project team suggests allocating this task to the secretary. They should be contacted to discuss their possibilities”
Contextual integration *Mean survey score: 3.44*	CP felt PROM need to be integrated in their EHRs, but also easy to share across organizations, but at this point impossible Resources (for project leader, IT, data analysis), external incentives (policy guidelines) and accreditation for learning were helpful Resources and leadership support varied, dependent on collaboration (and reimbursement) structure of OCNs and size of individual practices	*Evaluation report, OCN3, CM:* “Working in a system accessible across practices is nice! It is a pity we have to log-in to an external system first, and that this system isn’t connected to you own EHR. That would make it way easier to use as it [external system] costs a lot of extra time” *PTM16, OCN2, OB*: “This [decision to stop at end of project] represents two points very clearly: the need for one EHR and the fact that we have had many startup problems in this project”
*Reflexive monitoring* *“Appraisal work to assess and understand the ways that the innovation affects them and others”*		
Systemization	Response rates and practice experiences with input from colleagues (directly and from survey), discussed in project team and QI sessions Some CP discussed (negative) experiences amongst each other, without sharing with the project leaders Some CP indicated they did not receive feedback on project results or adaptations and felt unheard in their struggle to integrate in workflow Each team planned an evaluation report to their OCN at 12 months	*PTM7, OCN2, report*: (PL) “The project team questions whether we generate enough patients with the current selection in patient groups […] In case of little patients filling out PROM, there is a large change we forget to discuss completed PROMs. The decision is to start with the current patient groups, then evaluate whether we see enough patients, and if needed per January expand with diabetes gravidarum patients”
Communal appraisal	CP’s and patient’s experiences were leading in evaluating PROM value Overall, the value of PROM for daily practice and QI were seen but did not way the extra workload due to IT issues and the burden of an external system	*Evaluation report, OCN3, CM*: “As midwifery practice, we perceived that the use of PROM could lead to women preparing more consciously for a visit. As CP, we experienced that we sometimes gained more information in visits with PROM than without. Hence, topics like pain, urine and stool problems were discussed more often”
Individual appraisal	Many CP appreciated value of group-level PROM results for QI Appraisal for individual-level PROM was various across CP: in general, more valuable to hospital CP than community midwives PROM were considered unsuitable for women with low health literacy/non-Dutch speaking, whom CP believed would most benefit	*Evaluation report, OCN3, CM/PL*: “The QI sessions were inspiring and binding for our OCN and really led to positive action points for the OCN. Many of the attending CP reported afterwards to be enthusiast about using this method”
Reconfiguration	Learning from practice experiences and other regions, adaptations were made in close collaboration with the IT system Appeared hard to improve IT functionality, allocate administrative tasks and PROM content/timing At the end, conditions for future restart were formulated in evaluation reports to their OCN management	*PTM7, OCN3, OB*: “I had expected that it [the IT system] would be more developed, the technique works quite difficult. This whole meeting was about IT, it’s disturbing it can’t be tackled now. It has to be easy, and at the moment, I don’t experience it that way. X (PL) and X (OBR) are constantly on top of it: that really takes an excessive amount of time”

*PROM* patient-reported outcome measures, *PREM* patient-reported experience measures, *CP* care professional, *VBHC* value-based healthcare, *ICHOM* International consortium of health outcome measurement (developer of PCB set), *OCN* obstetric care network, *IT* information technology, *QI* quality improvement, *T5* time point 5 for measurement of patient-reported domains of the PCB set (6 months postpartum), *PTM* project team meeting, *CM* community midwife, *HM* hospital midwife, *OB* obstetrician/gynecologist, *OBR* obstetrics/gynecology resident, *PL* project leader (of local implementation team)

^a^PROM includes PROM and PREM in this table

^b^Rated on a 5-point Likert scale: a higher score indicates a more positive attitude

#### Coherence: sense-making

As terminology like PROM/PREM and VBHC often appeared abstract at the start, hearing experiences directly from participants of earlier regions helped to gain understanding of practical aspects. This was arranged both across and within OCN enhancing *differentiation* and *individual specification*. Care professionals early recognizing the potential benefits of PROM/PREM contributed to *internalization* and willingness for implementation. The ability to incorporate patients’ voice in QI appeared their main driver, so they were enthused by the QI sessions. Although some experienced that individual-level PROM/PREM supported time-efficiency and personalized care by discussing important rather than all topics, care professionals felt they needed more exposure to these benefits for sustained *internalization*.

#### Cognitive participation: relational work

Formally appointed local project leaders mainly drove *initiation*, particularly if this was a clinician from a participating practice with OCN management support (both in resources and vision). Project team members representing each participating practice and discipline could engage colleagues, reflect on practical challenges, and establish possible solutions. *Initiation* by key-participants was facilitated by action researchers’ activities (e.g., share experiences and materials, participate in identifying and solving issues, practical support) and by the one-year implementation period, making them feel able to try PROM/PREM without being ‘stuck’ to them. Whether local key-participants drove *initiation* or relied on the action researcher, depended on the level of ownership felt by local project teams. In-clinic support from key-participants and action researchers was most important for *enrolment* of other care professionals, since training reached a minority: 22% (22/99) of survey respondents had used support or training. *Enrolment* was harder in large practices, as care professionals felt less influence on the decision (or had little knowledge of the reasons) to participate. Care professionals differed in their feeling of PROM/PREM being a *legitimate* part of their role*,* which could be supported by positive practice experiences or those of colleagues. Additionally, *enrolment and legitimation* appeared to improve by the QI sessions, where valuable interprofessional conversations led to concrete improvement actions. However, care professionals’ positive expectations and involvement decreased over time by enduring IT issues and low exposure to benefits. At the end, (key) participants kept support for the potentials of PROM/PREM for VBHC and formulated future strategies for sustainable *activation*.

#### Collective action: operational work

Discussed in 92% of project team meetings (36/39), feasibility issues dominated the implementation process and impaired workflow integration (i.e., *interactional workability)*. Key-participants’ and action researchers’ time and efforts mainly went into getting the IT system working and supporting users (care professionals and patients) in operational work. Project teams experienced a vicious circle of poor-usable IT and not building up workflow routine: their attempts to increase routine, like expanding patient groups, were withheld by IT issues and concurrent time investment. The IT system affected participants’ confidence in the innovation (i.e., *relational integration*), especially the inability to improve or solve issues in time. Also, reliability of PROM/PREM results was questioned, because care professionals experienced varying clinical relevance of alerts, inappropriate timing, unsuitable answer options and, at group-level, numbers were too small. Most care professionals expressed confidence about discussing PROM/PREM, but the challenging part of *skill set workability* was allocating all tasks appropriately, for example ensuring that individual-level PROM/PREM were discussed across participating practices. To solve this, allocating a principal care provider to discuss PROM/PREM was opted by care professionals, both to keep overview of which responses had been discussed, as to gain most value from that conversation in a trusted relationship.

#### Reflexive monitoring: appraisal work

Facilitated by action researchers, project teams continuously reflected on (*systemization*) and tried to refine (*reconfiguration)* processes to improve implementation, like standard phrases to report PROM/PREM conversations to decrease administration burden. *Reconfiguration* was easier for smaller practices, such as temporarily collect T3 (maternity week) on paper to increase response rates. However, limited adaptability was experienced for several reasons: IT suppliers’ inability to improve, time constraints and the PCB set’s international origin. Key-participants’ evaluation reports stated *reconfigurations* needed for future restart and sustained implementation. For individual use, PROM/PREM should be easily accessible for patients and professionals, with EHR-integration across the network. For QI with group-level data, essential aspects were data analysis and visualization (provided by the researchers during the action research project) and linking PROM/PREM to clinical outcomes.

## Discussion

In this mixed-method evaluation of PROM/PREM implementation in the context of care networks, the use of PROM/PREM was found to be acceptable and appropriate but not feasible in daily practice, mainly due to IT issues and time constraints. Hence PROM/PREM implementation did not sustain, but their potentials for VBHC fitted professionals’ motivation and strategies for their future adoption were formulated in all OCN. In line with previous evidence [10, 11], our findings affirm the value of individual-level PROM/PREM for clinical care perceived by professionals and emphasize the need for workflow integration. Based on participants’ and researchers’ reflections on the re-adjusted, co-created implementation strategy, recommendations for PROM/PREM implementation across care networks were formulated in end-evaluations and summarized in Table 4. To embed these recommendations, an iterative approach is key to adjust to local context.

**Table 4:** Recommendations for PROM/PREM implementation across care networks

Aspect	Recommendations
PROM/PREM content	Individualize questionnaires: text field to elaborate on answers given
	Local adaptations to complement clinical workflow
	Ongoing PCB set governance based on implementation experiences in international collaboration
Training and support	Implementation support available in clinic
	Allocation of administrative staff
	PROM/PREM expert and clinical leader to drive implementation
	Learning directly from experiences in other regions
	Continuously inform CP and patient of primary purpose
Network collaboration	Case manager to discuss PROM/PREM for continuity across providers
	Infrastructure for data exchange across different providers/EHRs
	Connective leadership to focus innovations
IT and resources	PROM/PREM access integrated in EHR (CP and patient)
	Sustainable funding for network collaboration to develop/arrange data exchange across different providers
	External incentives (policy guidelines and protocols; time and accreditation for learning)

*CP* care professional, *PCB set* pregnancy and childbirth outcome set, *PROM* patient-reported outcome measures, *PREM* patient-reported experience measures, *EHR* electronic health record

Despite tailoring the strategy to our pre-implementation analysis amongst patients and care professionals and further adaption of implementation activities during each action research cycle [8, 28], the feasibility of integrating PROM in practice was lower than expected, largely explainable by poor usability of the IT system chosen at start. Of the numerous PROM/PREM capture systems developed in the past years, most were designed for single center settings or group-level, anonymous use only [11, 39, 40]*.* Besides healthcare systems with a shared EHR [41], successful system-wide PROM collection with direct visualization for individual-level use in clinic has proven challenging to realize and was only recently described and developed in a Welsh national program [42]. To support PROM/PREM implementation and network collaboration for patient-centered care, there is a need for PROM/PREM integration into EHRs and, moreover, infrastructures for cross-EHR data exchange [43]. Structural financial support for their development and governance should be explored, as most network collaborations are temporarily funded which undermines adoption, feasibility, and sustainability [4, 11, 44].

Previous PROM/PREM implementation strategies, both at the individual and group level, often emphasize the selection of PROMs and the challenge of involving care professionals [5, 11, 45]. Although we acknowledge their importance, most care professionals in our study already demonstrated a positive attitude towards PROM/PREM at start, reflected in good coherence and cognitive participation and consistent with previous findings [22, 41]. They were keen to learn from previous experiences and motivated by the prospective of patient-centered QI with group-level PROM/PREM, which fueled their efforts for individual-level implementation as well. In the current healthcare landscape with professional shortage and high turnover, care professionals’ work pleasure might be one of the most valuable benefits of PROM/PREM [46, 47]. Despite feasibility challenges and IT issues, key participants’ threshold to adopt such complex implementation was lowered by the iterative approach that gave space to ‘try out’ and adapt to local context, which enabled them to get acquainted with PROM/PREM and their potential for VBHC. Concurrently, other care professionals felt demotivated and overruled by management when unaware of the reasons to participate in such implementation and driving their workload even higher. So new initiatives should be carefully selected and coordinated across care networks, where an iterative and participative approach to implementation can provide space for early adopters’ energy, sharing practice experiences to engage others, and fine-tuning to local context.

The integrated care context affected implementation not only by challenges in IT infrastructure, fragmented leadership and allocation of costs, but also in consistency of discussing individual-level PROM/PREM results across care transitions. To ensure that individual-level results were discussed, care professionals opted to allocate a principal care provider, arguing that a conversation about the topics would gain most value in a trusted relationship, similar to a solution to improve continuity of perinatal care in general [48]. However, the issues arising from network-broad implementation are lacking in current PROM/PREM implementation frameworks and strategies [9, 10]. Further research within real-life projects should identify and address barriers and enablers for innovation across organizational boundaries. That way, innovations can improve value of care for individuals and overall care performance from patients’ perspective.

Reflecting on the action researchers’ role, many similarities were seen with the facilitator role described by Roberts in the iPHARIS framework [49]. Similar to their findings, our action researcher was a crucial enabler for implementation, providing an external view with expert knowledge to identify and solve emerging issues in practice, especially in collaboration with the local project leader. Additionally, participating in all regions resulted in overview, expertise and sharing previous lessons in new regions. However, the tension between guidance in problem solving and doing the work to fit local workflow was present in our projects as well: in some regions, the PROM/PREM workflow never became completely independent of the action researcher. Across OCN, the level of implementation ownership of the project teams varied, which could partly be explained by existing collaboration mechanisms and integrated reimbursement in some OCN.

As called for in recent literature [10, 16], this study substantially contributes to the understanding of care professionals’ real-life experiences and challenges for PROM/PREM implementation, specifically addressing the integrated care context in a realistic range of collaborating organizations. In the mixed-methods design, consistency in data from different sources and methods strengthened our findings. Also, our data collection and analyses were supported by widely used implementation science theories and their validated instruments. The iterative, participatory action research approach enabled in-depth understanding of implementation activities, processes and outcomes, which contributes to the transferability of findings. An important limitation of our study was that we did not invite patients to the evaluation of the implementation process and outcomes, except indirectly via care professionals. We did explore patients’ experiences with individual-level use in another study along a national pilot with the PCB set [50], while the current project focused on the (organizational challenges of) implementation. In next action cycles, patients should be certainly involved. Here, special attention should go to women with low health-literacy and language barriers, who are prone to be neglected by PROM/PREM, to prevent existing health inequities becoming even larger [51]. Besides providing digital support and translating questionnaires, solutions to involve these women should be sought outside the idea of questionnaire completion. In thinking of solutions, research methods should be embraced that centralize patients and local opportunities (e.g. linkage to primary care, community-based solutions) [52, 53]. Another limitation of our study is that the IT-system used appeared such a major barrier to implementation, that other factors might have been undervalued. Selection bias of both early adopter OCN and professionals is likely to have enhanced a positive attitude towards PROM/PREM. We attempted to reach professionals broader by inviting the whole OCN for QI sessions and the survey, which had a short version for indirectly involved professionals. Lastly, the COVID-19 outbreak has probably influenced care professionals’ willingness and ability to adopt a new way of working, affected implementation planning (e.g., paused, postponed) and restricted study activities to online contacts with minimal field work.

### Conclusion

Although implementation did not sustain, network-broad PROM/PREM use in clinic and for QI matched professionals’ motivation for patient-centered care. This study provides recommendations to implement PROM/PREM meaningfully in practice, in ways that support professionals in their drive towards patient-centered care by efficient, person-centered assessment of patients’ wellbeing. For PROM/PREM to fulfill their potential for VBHC, our work highlights the need for sustainably funded technology infrastructures that communicate across healthcare tiers, as well as an iterative and participative approach to refine their complex implementation to local contexts.

## Supplementary Information

**Additional file1**. **Figure S1**: Patient-reported domains and timeline of their measurement (PCB set). **Table S1**: Normalization Process Theory (NPT): mechanisms, subconstructs and assessment. **Table S2**: Full statements of the implementation survey administered to obstetric healthcare professionals to evaluate the implementation of the PCB set. **Table S3**: Topic guide statements for focus groups in care professionals attending QI session. **Figure S2**: Stacked-bar graphs of survey outcomes.

## Data Availability

The datasets used and/or analyzed during the current study are available from the corresponding author on reasonable request.

## Abbreviations

ICHOML: International Consortium for Health Outcomes Measurement
OCN: Obstetric care networks
PCB set: Pregnancy and childbirth patient-centered outcomes set
PREM: Patient-reported experience measures
PROM: Patient-reported outcome measures
QI: Quality improvement
HER: Electronic health record
