# Supplementary material for: PROMs and PREMs in routine perinatal care: mixed methods evaluation of their implementation into integrated obstetric care networks
Source: J Patient Rep Outcomes. 2023 Mar 9;7:26. doi: 10.1186/s41687-023-00568-w (PMC9998006; doi:10.1186/s41687-023-00568-w)

**Additional File 1**

*Manuscript Title:*

PROMs and PREMs in routine perinatal care: mixed methods evaluation of their implementation into integrated obstetric care networks.

*Content Supplementary information:*

**Figure S1.** Patient-reported domains and timeline of their measurement (PCB set).

**Table S1.** Normalization Process Theory (NPT): mechanisms, subconstructs and assessment

**Table S2.** Full statements of the implementation survey administered to obstetric healthcare professionals to evaluate the implementation of the PCB set.

**Table S3.** Topic guide statements for focus groups in care professionals attending QI session.

**Figure S2.** Stacked-bar graphs of survey outcomes.

**Figure S1.** Pregnancy and Childbirth outcome set: patient-reported domains and timeline of their measurement

Figure adapted from Nijagal et al [1]. *PROM,* patient-reported outcome measure. *PREM,* patient-reported experience measure. The original outcomes set has been translated to the Dutch language and setting by Laureij et al [2]. For a full description of measurement instruments and scoring systems per domain, refer to Depla et al [3].
*T1*, time point 1. *T2*, time point 2. *T3*, time point 3. *T4*, time point 4. *T5*, time point 5.

1. Nijagal MA, Wissig S, Stowell C, et al. Standardized outcome measures for pregnancy and childbirth, an ICHOM proposal. BMC Health Serv Res. 2018;18(1):953. doi:10.1186/s12913-018-3732-3

2. Laureij LT, Been J V., Lugtenberg M, et al. Exploring the applicability of the pregnancy and childbirth outcome set: A mixed methods study. Patient Educ Couns. 2020;103(3):642-651. doi:10.1016/j.pec.2019.09.022

3. Depla AL, Ruiter ML de, Laureij LT, et al. Patient-Reported Outcome and Experience Measures in Perinatal Care to Guide Clinical Practice : Prospective Observational Study Corresponding Author : J Med Internet Res. 2022;24(7):e37725. doi:10.2196/37725

**Table S1.** Normalization Process Theory (NPT): mechanisms, subconstructs and assessment

| **Mechanism** | **Subconstruct** | **Definition** | **Assessment methods in this study** |
| --- | --- | --- | --- |
| Coherence (sense making) | Differentiation | Participants see how (the set of new practices)^a^ differs from usual ways of working | observation^b^, qualitative methods^c^ |
|  | Communal specification | Staff in this organization have a shared understanding of the purpose of (the set of new practices) | observation, qualitative methods |
|  | Individual specification | Participants understand how (the set of new practices) affects the nature of my own work | observation, qualitative methods |
|  | Internalization | Participants construct the potential value of (the set of new practices) for my work | observation, qualitative methods |
| Cognitive participation (relational work) | Initiation | Key people are driving (the set of new practices) forward and get others involved | survey^d^, observation, qualitative methods |
|  | Enrolment | Participants (re)organize themselves to contribute to the work involved in (the set of new practices) | survey, observation, qualitative methods |
|  | Legitimation | Participants believe it’s right to be involved in (the set of new practices) and that they can make a valid contribution to it | survey, observation, qualitative methods |
|  | Activation | Participants continue to support (the set of new practices) | survey, observation, qualitative methods |
| Collective action (operational work) | Interactional workability | Participants execute the tasks of the (the set of new practices) and integrate them into their existing work | survey, observation, qualitative methods |
|  | Relational integration | Participants build accountability and maintain confidence in each other as they use (the set of new practices) | survey, observation, qualitative methods |
|  | Skill set workability | The work of (the set of new practices) is allocated to the right persons who receive sufficient training | survey, observation, qualitative methods |
|  | Contextual integration | The work of (the set of new practices) is adequately supported by management and resources | survey, observation, qualitative methods |
| Reflexive monitoring (appraisal work) | Systemization | Participants have access to information about the effects of (the set of new practices) | observation, qualitative methods |
|  | Communal appraisal | Participants collectively agree that (the set of new practices) is worthwhile | observation, qualitative methods |
|  | Individual appraisal | Participants individually value the effects that (the set of new practices) has had on their work | observation, qualitative methods |
|  | Reconfiguration | Participants modify their work with (the set of new practices) based on their appraisal of the (the set of new practices) | observation, qualitative methods |

^a^ *The set of new practices:* in this project defined as the use of PROM/PREM in individual care and quality improvement ^b^ *Observations:* performed along a checklist with NPT subconstructs while participating in implementation activities (i.e., project team meetings, kick-off sessions, QI sessions and two-weekly reflection logbook). ^c^ *Qualitative methods*: NPT subconstructs were embedded in coding schemes of all qualitative data (i.e., open-ended survey answers, transcriptions, observation reports, reflection logbook, and naturally occurring documents) ^d^ *Survey:* measured with the validated NoMAD (Normalization MeAsure Development) instrument.

**Table S2.** Full statements of survey administered to care professionals to evaluate the implementation of the PCB set including the NoMAD (a), MIDI (b), and extra evaluation (c).

| **(a)** | **Baseline** |  |  |
| --- | --- | --- | --- |
| **No.** | **Label** | **Survey A/B^a^** | **Item** |
| B1 | Region | Both | In which OCN do you work? |
| B2 | Professional role | Both | What is your profession within your OCN? |
| B3 | Working experience | Both | How many years of experience do you have *a) in this profession; b) in this OCN?* |
| B4^b^ | Implementation role | Both | What is your role in the implementation of [the intervention]? |
| B5 | Start | A | Has implementation of [the intervention] started in you OCN yet? |
| B6^c^ | Use | A | Are you using [the intervention] yourself? |
| **(b)** | **NoMAD^d^** |  |  |
| **No.** | **Subconstruct** | **Survey A/B** | **Item** |
| CP1 | Initiation | Both | There are key people who drive [the intervention] forward and get others involved |
| CP2 | Legitimation | Both | I believe that participating in [the intervention] is a legitimate part of my role |
| CP3 | Enrolment | Both | I’m open to working with colleagues in new ways to use [the intervention] |
| CP4 | Activation | A | I will continue to support [the intervention] |
| CA1 | Interactional workability | A | I can easily integrate [the intervention] into my existing work |
| CA2^e^ | Relational integration 1 | A | [The intervention] disrupts working relationships |
| CA3 | Relational integration 2 | Both | I have confidence in other people’s ability to use [the intervention] |
| CA4^c^ | Skill set workability 1 | A | Work is assigned to those with skills appropriate to [the intervention] |
| CA5 | Skill set workability 2 | A | Sufficient training is provided to enable staff to implement [the intervention] |
| CA6 | Contextual integration 1 | A | Sufficient resources are available to support [the intervention] |
| CA7 | Contextual integration 2 | A | Management adequately supports [the intervention] |
| GN1^c,f^ | Past normality | A | When you use [the intervention], how familiar does it feel? |
| GN2^c,f^ | Current normality | A | Do you feel [the intervention] is currently a normal part of your work? |
| GN3^f^ | Future normality | A | Do you feel [the intervention] will become a normal part of your work? |
| **(c)** | **MIDI** |  |  |
| **No.** | **Subconstruct** | **Survey A/B** | **Item** |
| U8^g,h^ | Personal benefit/drawback | Both | [The innovation] contributes to *recognize symptoms and changes in them / insight in what matters to my patient / set priorities for the conversation / the shared decision-making process / the relationship with my patient / insight in the results of care I deliver / quality improvement of care pathways in the OCN / allocate who provides which care in the OCN*  ^‡^ Possible drawbacks of [the innovation]: *I have too little information, knowledge, or experience to use it / it takes too much time in my daily work / I don’t believe it benefits perinatal care / completing the questionnaires is too burdensome for my patients / the costs are too high for the OCN or my organization / data from the questionnaires are subjective, thus unreliable* |
| U9^g,h^ | Outcome expectations | Both | My patient will … *have a lower threshold to raise issues* / *be better prepared to her care trajectory / be empowered in her care trajectory / have more insight in relevant outcomes of care and choice information /* *receive more personalized care that fits what matters to her / other outcome expectations*^i^ |
| U12 | Client cooperation | A | Clients will generally cooperate if I use [the innovation]. |
| U13 | Social support | A | I can count on adequate assistance from my colleagues if I need it to use [the innovation]. |
| U15^h^ | Subjective norm:  motivation to comply | A | When it comes to working in accordance with [the innovation], to what extent do you comply with the opinions of *your direct colleagues / your local OCN / your* *regional obstetric collaboration / pregnant women / the national obstetric care collaboration*? |
| U16^h^ | Self-efficacy | A | Should you wish to do so, do you think you can *interpret answers to the PCB set questionnaires and discuss them with pregnant women / act appropriately on answers to the PCB set questionnaires if needed / improve the quality of care in the OCN with group-level data from the PCB set questionnaires?* |
| O26 | Unsettled organization | A | Are there, in addition to the implementation of [the innovation], any other changes in the organization affecting the implementation of the innovation now or in the foreseeable future (reorganization, merger, cuts, staffing changes, other innovations)? |
| O28 | Performance feedback | B | In my organization, feedback is regularly provided about progress with the implementation of [the innovation]. |
| **(d)** | **Extra evaluation** |  |  |
| **No.** | **Subconstruct** | **Survey A/B** | **Item** |
| E1^i^ | Extra evaluation | A | What do you need to become able to do this?*^j^* |
| E2^k^ | Extra evaluation | A | Which supportive materials do / did you use when working with [the innovation]? |
| E3 | Extra evaluation | A | Which aspects are important to embed [the innovation] in your daily work? Please indicate to what extent the following aspects are important to you: *Ease of use / Costs (capture and governance) / Time efficiency / Dashboard per client (compare over time and with group level results) / Embed in existent IT systems (EHR etc.) / Reliability or clinical relevance of answers / Adjustment to current registries / Other aspects*^i^ |
| E4^b^ | Extra evaluation | Both | To what extent do you know about the [intervention]? |

Statements were measured on a 5-point Likert scale of 1 (strongly disagree) to 5 (strongly disagree). ^a^ Based on their answer to question B4, respondents were led to survey A or B: *Survey A* for participants with direct involvement in implementation (i.e., participation in the implementation team or using the PROM in practice)*. Survey B* for participants with indirect involvement in implementation. ^b^ Multiple choice. ^c^ Optional: only asked if implementation had started (baseline question B5). ^d^ The additional response option of the NoMAD instrument to indicate if a statement was applicable was not included. ^e^ Conversed for analysis since this was a negative statement. ^f^ Measured on a 0 (not at all) to 10 (completely) visual analogue scale. ^g^ Researchers had to state (U8) concrete expected benefits/drawbacks for the user and (U9) intended objectives of the innovation. ^h^ All phrases in italic that are separated by a dash were questioned in separate statements and averaged to provide a single subconstruct score for analysis. ^i^ Open-ended. ^j^ Optional: dependent of U16 (self-efficacy), only asked in case of negative answer to a specific task. ^k^ Checkboxes with dichotomous scale ('yes'/'no').
*No.*, abbreviated construct name plus item number. *NoMAD*, Normalization MeAsure Development instrument. *B,* baseline. *GN,* global normalization. *CP,* cognitive participation. *CA,* collective action. *MIDI*, Measurement Instrument for Determinants of Innovations. *U,* user. *O,* organization. *E,* extra evaluation. *[the innovation]*, capture and use of the PCB set’s questionnaires. *OCN,* obstetric care network.

**Table S3.** Topic guide (statements) for focus groups in care professionals attending QI sessions

| **Statement** | **Focus group #** |
| --- | --- |
| [The intervention] has more added value for patient and care professionals in the consultation room, than for learning and improving as OCN. | 1,2^a^ |
| A good accessible IT system for the questionnaires would certainly have led to successful implementation. | 2 |
| As OCN, we learn and improve already with outcome information (enough). | 1 |
| We will continue these learning and improvement sessions (QI session): if not with PROM/PREM-data, then with other data available about our OCN (e.g., from the national registration). | 2 |
| Learning and improving always comes on top of our normal/daily work. | 1,2 |
| Midwives and obstetricians are the most important stakeholders for learning and improving in an OCN. | 1,2 |
| As OCN, we are able to carry out joint improvement actions. | 1,2 |

Participants could ‘vote’ yes/no to the statements to facilitate discussion about the statements. *^a^ Focus group 1* was held in phase 2 (month 6-9) and *focus group 2* in phase 3 (month 9-12)*.*

*[The intervention],* capture and use of the PCB set’s questionnaires. *OCN,* obstetric care network. *QI,* quality improvement.

**Figure S2**. Stacked-bar graphs of survey outcomes

*Supplementary figure 2a.* NoMAD items


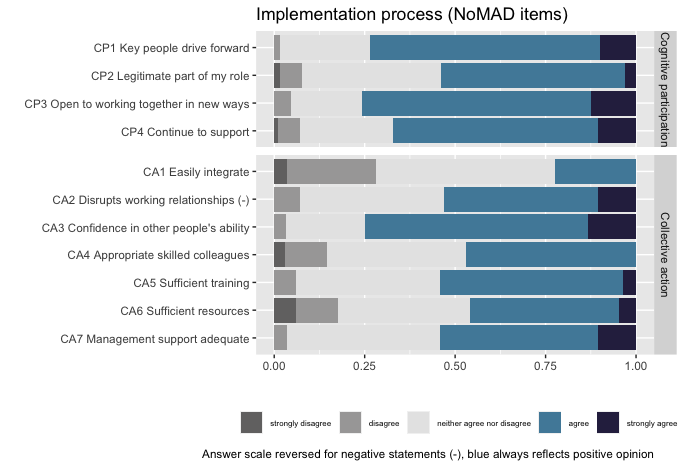


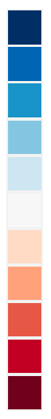
**
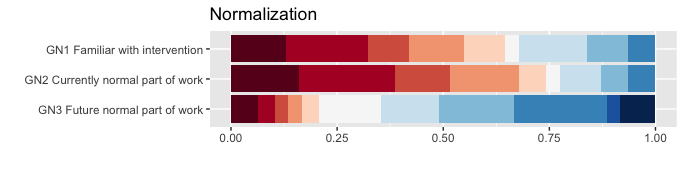
**

Answer scale 0 (red) to 10 (blue)

*Supplementary figure 2b.* MIDI items


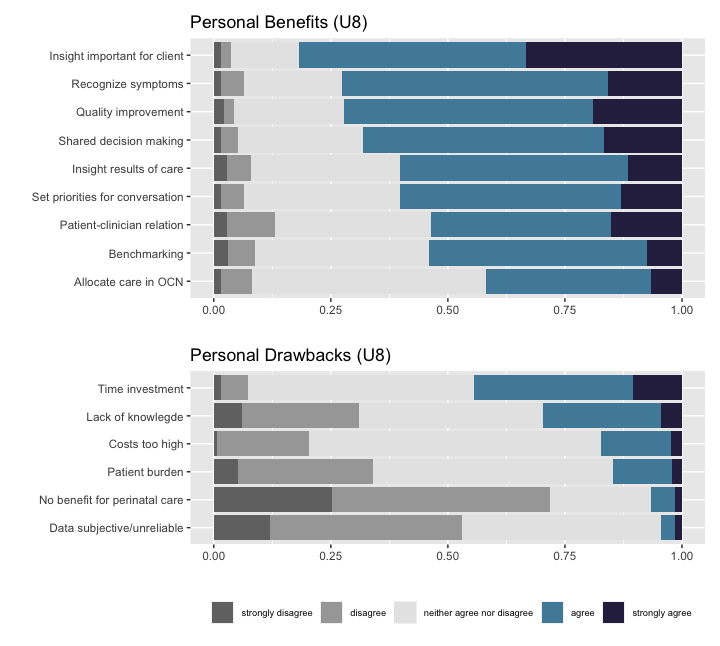


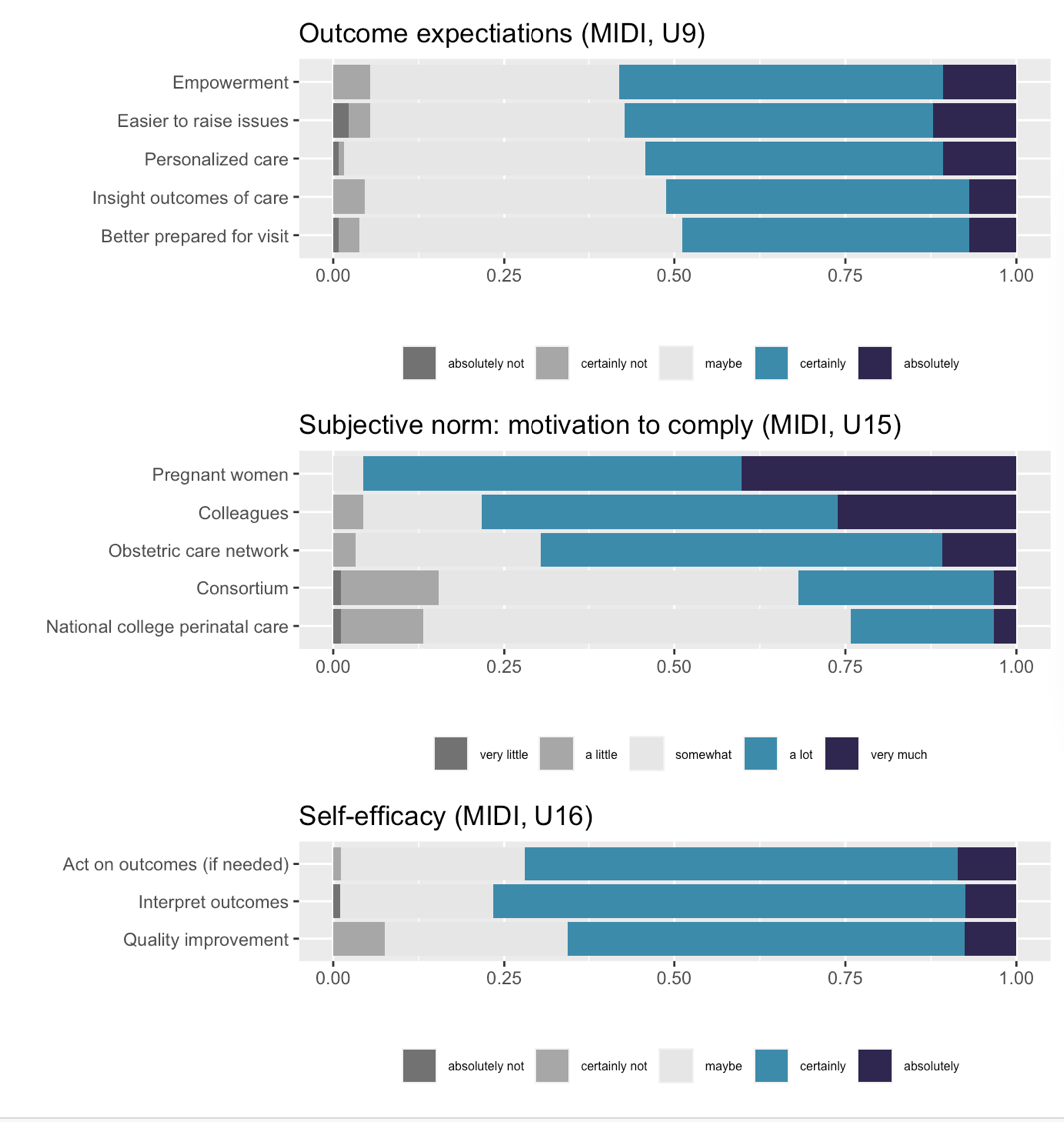


Continued: S*upplementary figure 2b.* MIDI items


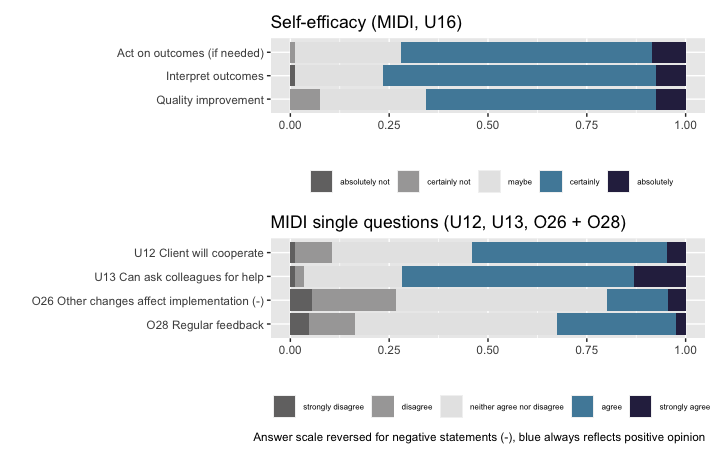


*Supplementary figure 2c.* EXTRA Questions


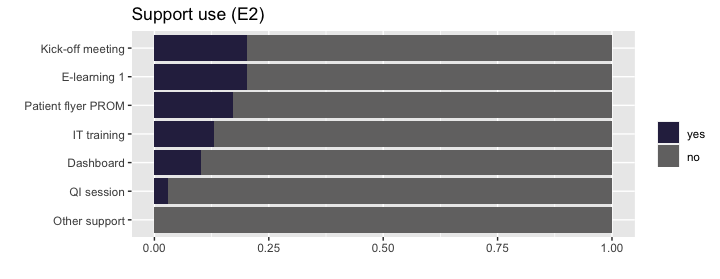


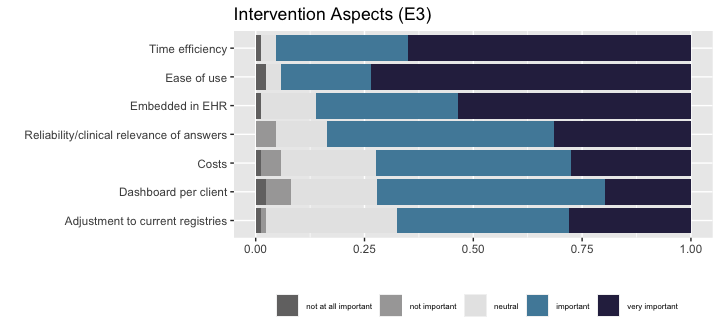


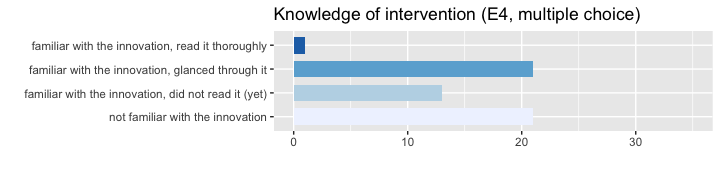

Supplement: Supplementary file 1 — Additional file1. Figure S1: Patient-reported domains and timeline of their measurement (PCB set). Table S1: Normalization Process Theory (NPT): mechanisms, subconstructs and assessment. Table S2: Full statements of the implementation survey administered to obstetric healthcare professionals to evaluate the implementation of the PCB set. Table S3: Topic guide statements for focus groups in care professionals attending QI session. Figure S2: Stacked-bar graphs of survey outcomes. [file 41687_2023_568_MOESM1_ESM.docx]
